# Prehabilitation, making patients fit for surgery – a new frontier in perioperative care

**DOI:** 10.1515/iss-2019-0017

**Published:** 2019-12-24

**Authors:** Charlotte J. L. Molenaar, Nicole E. Papen-Botterhuis, Florian Herrle, Gerrit D. Slooter

**Affiliations:** Department of Surgical Oncology, Máxima MC, Veldhoven, the Netherlands; Department of Medical Oncology, Máxima MC, Veldhoven, the Netherlands; Department of Surgery, University Medical Centre Mannheim, Medical Faculty Mannheim, University of Heidelberg, Heidelberg, Germany; Department of Surgical Oncology, Máxima MC, 5500MB, Veldhoven, the Netherlands, E-mail: prehab.resurge@mmc.nl

**Keywords:** functional capacity, multimodal, perioperative care, prehabilitation, surgical complications

## Abstract

Optimizing a patients’ condition before surgery to improve the postoperative outcome can be achieved by using prehabilitation; preoperative interventions focusing on modifiable risk factors to improve the physical, nutritional, and mental status of the patient. A multimodal, multidisciplinary approach induces a synergistic effect between the various interventions and affects the outcome postoperatively. While awaiting higher-quality evidence, the worldwide implementation of prehabilitation programs has started, resulting in a true revolution in perioperative care.

## Introduction

“Prehabilitation”, “Fit 4 Surgery”, “Fit 2 Fight”, “pre-rehabilitation”, and “better in – better out” are all expressions of an identical revolutionary thought to optimize the patients’ condition before surgery. Prehabilitation is defined to include assessment of physical, nutritional, and psychological status to determine baseline functional capacity, identify impairments, and intervene in order to improve the patients’ preoperative functional reserve prior to treatment [1], [2]. The interventions address modifiable risk factors with the intention to improve the outcomes of (cancer) treatment [2]. Both short-term outcomes of treatment as well as long-term behavioral changes can be altered [3]. It leads to an improved functional capacity [4], [5], [6], [7], improved nutritional [8], [9] and mental status [9], [10], reduction of complications [11], [12], [13], faster recovery [7], [14], [15], a reduced length of stay in the hospital [16], an improved quality of life [10], [17], and potentially a reduction in costs. While the evidence for improvement of clinical outcomes is growing, prehabilitation is at the threshold of worldwide implementation and already included in guidelines [18], [19], [20].

A growing interest in the postoperative outcome (e.g. quality of life) is the result of rising life expectancy and increasing survival rates of cancer [21], [22]. The fact that metabolic and behavioral risk factors are rising and will continue to do so [23] makes it a necessity for healthcare professionals to raise awareness and emphasize the role of the patient. Lifestyle changes are complex; however, patients are more willing to change behavior positively when facing major surgery [3].

Innovations and technologies in the past 50 years have contributed to the reduction in death rates [22]. Perioperative care is one of the aspects of healthcare, where many developments significantly improved the outcome. Laparoscopy, for instance, has become the golden standard for various surgical procedures due to the positive results. It is also considered as the integral core of Enhanced Recovery After Surgery (ERAS) programs [24], [25]. ERAS programs were introduced in the late 1990s. The principles of ERAS are based on the knowledge that many of the negative effects of major surgery (e.g. loss of muscle mass and body weight as well as a reduced resistance to infection) can be reduced by attenuating the surgical stress response with perioperative interventions [26]. Interventions such as anemia correction, assessment of the risk of postoperative delirium, perioperative pain management with a multimodal opioid-sparing analgesia regime, and anti-ileus management are essential components of an ERAS program [27]. The ultimate purpose is to minimize organ dysfunction postoperatively and enhance rehabilitation [28].

However, despite all of the above, surgery is still related with postoperative morbidity with complications rates varying from 18.4% in non-small cell lung cancer surgery to 65% in esophageal cancer surgery in The Netherlands in 2017 [29]. Postoperative complications result in an increased length of stay in the hospital, readmissions, and elevated healthcare costs, impact patient functioning and quality of life, and have possible implications on mortality [30]. The number of preoperative modifiable risk factors is associated with the risk of severe complications [31]. Furthermore, there is a strong correlation between the number of postoperative complications and the patients’ preoperative functional capacity and nutritional and mental status as determined by lifestyle and behavioral habits such as smoking and drinking [32], [33], [34], [35]. Therefore, further progress in improving perioperative care is desirable.

**Figure 1:**
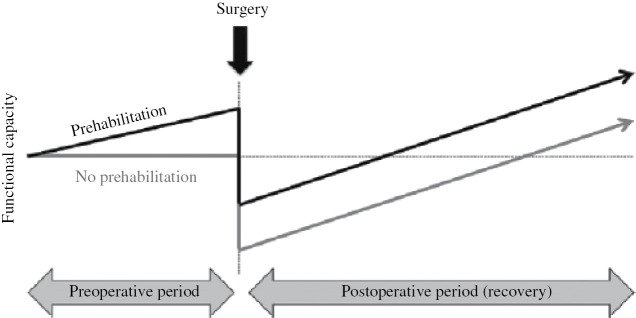
Concept of prehabilitation. Theoretic model of surgical prehabilitation based on the concept of increasing functional capacity before surgery. Adapted with permission from Carli F, Zavorsky GS. Optimizing functional exercise capacity in the elderly surgical population. Curr Opin Clin Nutr Metab Care 2005; 8: 25.

Until recently, efforts to improve recovery after major surgery have primarily focused on intra- and postoperative care [19]. The postoperative period is associated with anxiety and depression, fatigue and weakness, lack of sleep, and anorexia. It is therefore not the optimal period to introduce interventions to accelerate recovery [36], [37]. Furthermore, by addressing the functional capacity preoperatively, the postoperative risk is attenuated [36]. The preoperative period, although limited to several weeks, may be the window of opportunity to improve the patients’ condition and facilitate persistent lifestyle changes [5], [38], [39]. That is where prehabilitation comes in place (Figure 1).

Preparing a patient for surgery can have different aspects and goals. In orthopedics, specific joints might be trained in strength and range of motion before, for example, hip replacement. Preoperative training will facilitate the recovery of joint function, leading to a shorter hospital stay [40]. In breast cancer surgery, preoperative exercise will have a positive effect on the recovery of the upper extremity functioning after mastectomy [41]. Preparing a patient for abdominal or thoracic surgery, however, is meant to improve the patients’ resilience against the impact of surgery, as even without complications there is a postoperative reduction of functional capacity [42], [43].

Prehabilitation should be multimodal. All pillars on its own affect the surgical outcome, both physically and psychologically. However, there is a synergistic effect of improving a patients’ physical condition, balancing and supplementing nutritional needs, altering mental status through psychological support, and cessation of smoking [44]. This multimodal approach seems unconditional, as exercise and dietary protein intake produce independent and additional effects on anabolism and muscle protein synthesis [45]. A positive mental status will enforce the motivation for training and eating, whereas studies with rodents imply that exercise recruits midbrain dopamine and striatal circuits involved in reward and stress resistance [46], thus improving mental status. Cessation of smoking is included because stopping, even shortly before an operation, will reduce the detrimental effect of the consequences of smoking [47], [48]. One could consider an extra pillar in prehabilitation: correction of other risk factors related to the disease such as anemia, which is present in about half of colorectal cancer patients. Preoperative anemia is associated with increased postoperative complications and decreased overall and disease-free survival in rectal cancer [33], [49]. The correction of the preoperative value is described in the context of ERAS [27] and additionally suggested to increase adherence to prehabilitation [36]. Polypharmacy could be addressed preoperatively, but at Máxima MC we consider both mentioned interventions as part of standard care and suggest, for example, iron supplementation when hemoglobin levels are <7 mmol/L (11.3 g/dL) as well as the involvement of the anesthetist as early in the oncological pathway as possible.

**Figure 2:**
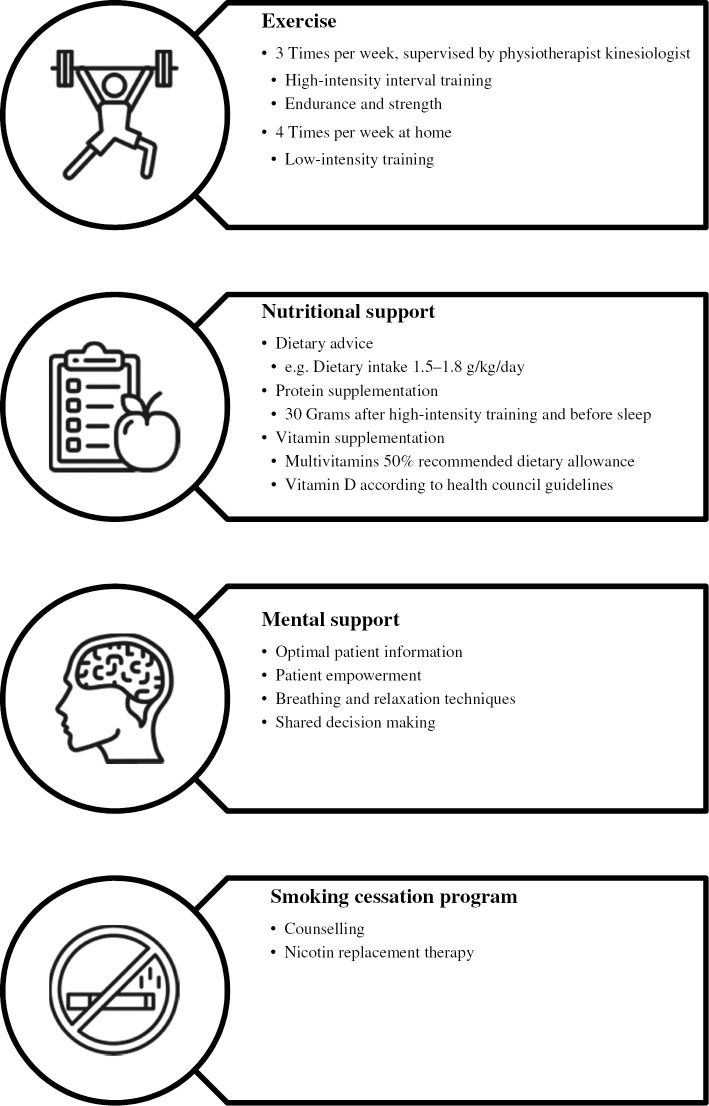
International prehabilitation protocol.

An international research consortium on prehabilitation, under guidance of professor Dr. Francesco Carli from McGill University Montréal, has created “best practice” for multimodal prehabilitation in colorectal cancer surgery in 2016. A four-pillar program consists of high-intensity interval training on endurance and strength, nutritional support with protein and vitamin supplementation, mental support, and a smoking cessation program (Figure 2). This program has been adapted in many clinical studies and subsidiary requests, also for diseases other than colorectal cancer populations.

**Figure 3:**
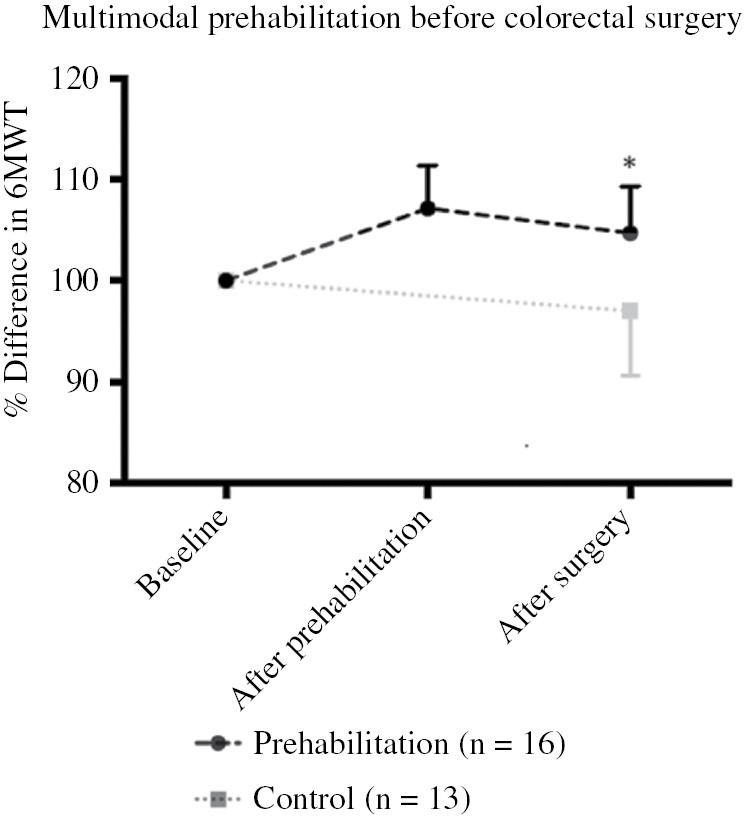
Pilot study on multimodal prehabilitation before colorectal cancer surgery. Patients who underwent a prehabilitation program of 4-weeks before colorectal cancer surgery showed progress after training and performed better 4-weeks postoperatively (p<0.05, analysis of variance) on functional capacity (6MWT) compared to controls who received standard preoperative care.

The research consortium is conducting the international PREHAB trial (NL58281.015.16) [50], which started in 2017. Some 714 participants undergoing resection for colorectal carcinoma are being randomized in either the control or the four-pillar multimodal prehabilitation program. Perioperative care is given according to ERAS guidelines. Eight hospitals in five different countries contribute to the study, but the final inclusion is not to be expected before the end of 2020. A nonrandomized feasibility study of the protocol was conducted at Máxima MC. Program evaluation revealed a high attendance rate (90%) and a high level of patient satisfaction. There were no adverse effects, and both strength and endurance were improved preoperatively. Figure 3 illustrates the increase in functional capacity [6-min walk test (6MWT)] after 4 weeks of prehabilitation. Four weeks after surgery, there was a significant difference compared to control patients. Forty percent of control patients were at their baseline functional capacity 4 weeks after surgery, whereas in the prehabilitation group 86% were at or higher than their baseline level (p<0.01) [15].

It might sound logical to improve modifiable risk factors in the period before surgery to reduce complications, but the implementation of prehabilitation needs true courage and management changes within each hospital [51]. Even believers will face managers only looking at the cost of prehabilitation and do not take into account the potential benefits of the reduction of complications and hospital stay. In a sense, they might be right as evidence is not strong yet and recommendations are still weak [19].

High-level evidence that multimodal prehabilitation will reduce complications and improve outcome must come from randomized studies. The research group of Dr. G. Martínez-Palli from the Hospital Clínic de Barcelona published a study on 144 randomized patients undergoing major abdominal surgery. Prehabilitation contained a motivational interview, high-intensity training, and promotion of lifestyle and physical activity. Sixty-five patients were evaluated. In the intervention group, aerobic capacity was enhanced and the number of patients with postoperative complications was reduced by 51%. Also, the rate of complications was reduced significantly [11]. The latest systematic reviews on prehabilitation in abdominal surgery described similar beneficial effects of prehabilitation but also stated the need for a well-designed randomized controlled trial to evaluate the positive effects in more detail and to identify suitable target populations [12], [13]. Several studies using hospital- or home-based prehabilitation for various surgical procedures have been or will be initiated [50], [52], [53], [54], [55]. We hope that more evidence will be available before the publication of the results of the international PREHAB study in colorectal cancer at the end of 2020, as we do not want to wait with broad implementation.

A potential negative aspect of prehabilitation could be a delay of surgery. The interval between referral and treatment, the so called “window of opportunity”, is limited. However, at least some extra time can be bought by optimizing the diagnostic pathway. Our personal experience is that the interval between referral and diagnosis as determined during a multidisciplinary team meeting can be as short as 1 week. At Máxima MC, the delay caused by the prehabilitation program in patients with colorectal surgery was limited to 1 week. The literature, however, states that a treatment delay of 56–62 days (>8 weeks) should not be detrimental for the patient with respect to long-term outcomes [56], [57]. A recent publication might be very persuasive, demonstrating improved disease-free survival after prehabilitation for stage III colorectal cancer surgery [58].

## International cooperation

In 2017, Professor Carli brought together a limited number of international experts on perioperative care and prehabilitation in Montréal, Canada. All aspects of multimodal prehabilitation were discussed and definitions were determined. The next convention, called the Second Prehabilitation World Conference, was held in Eindhoven, The Netherlands, in 2018, hosting more than 450 delegates from 22 countries. During the 2018 conference, the International Prehabilitation Society was initiated (www.prehabsociety.com). The society, together with the Royal College of Anaesthetists, organized the Third World Conference at the British Museum in London. The program was sold out and hosted more than 700 surgeons, anesthetists, (specialized) nurses, physical therapists, nutritionists, sports physicians, psychologists, urologists, orthopedists, and representative from other disciplines. A guideline on prehabilitation for cancer patients developed on behalf of Macmillan cancer support together with the Royal College of Anaesthetists and the National Institute of Health Research was presented at this congress. This document proposes a framework for implementation [59].

The 2020 event will be held in Barcelona, Spain, and the 2021 event is scheduled in Melbourne, Australia.

The International Prehabilitation Society wants to bring together all stakeholders involved in making patients fit for surgery. Although we believe in high-intensity, multimodal prehabilitation, any initiative to improve a patients’ physical, nutritional, and mental status before surgical or medical therapy is considered of value. We want to share knowledge and protocols while gathering the evidence that is needed for the broad implementation of prehabilitation. The International Prehabilitation Society has initiated a communication platform for members and nonmembers and encourages every country to create a national chapter and website to facilitate national cooperation. Healthcare professionals who want to contribute to the German Chapter are kindly asked to contact Dr. Florian Herrle (florian.herrle@umm.de).

## Conclusion

Multimodal prehabilitation before surgery increases the patients’ physical, nutritional, and mental status before surgery. There is growing evidence that prehabilitation leads to a reduction of complications and a faster recovery after surgery. Although it has already been adopted in several new guidelines, we are still at the threshold of implementation. The question remains: Should we offer our patients the chance to get fit for surgery now or do we need to wait for more evidence?
