# Supplementary material for: Prehabilitation, making patients fit for surgery – a new frontier in perioperative care
Source: Innov Surg Sci. 2019 Dec 24;4(4):132–8. doi: 10.1515/iss-2019-0017 (PMC8059351; doi:10.1515/iss-2019-0017)
Supplement: Supplementary file 1 [file iss-04-20190017-s001.pdf]

Reviewer Assessment

Charlotte J.L. Molenaar, Nicole E. Papen-Botterhuis, Florian Herrle and Gerrit D. Slooter*

Prehabilitation, making patients fit for surgery – a new frontier in perioperative care

https://doi.org/10.1515/iss-2019-0017
Received December 9, 2019; accepted December 9, 2019

***Corresponding author: Gerrit D. Slooter,** Department of Surgical Oncology, Máxima MC, 5500MB, Veldhoven, the Netherlands, E-mail: g.slooter@mmc.nl, prehab.resurge@mmc.nl

Editors’ Comments to Original Submission and Final Decision

The contribution is not a scientific paper or a formal review, but it presents the current situation and findings on the subject of prehabilitation. It is of high quality and therefore it should be published in the current state.

Open Access. © 2019 Molenaar C.J.L., et al., published by De Gruyter. This work is licensed under the Creative Commons Attribution 4.0 Public License.
